# Intrinsic proteotoxic stress levels vary and act as a predictive marker for sensitivity of cancer cells to Hsp90 inhibition

**DOI:** 10.1371/journal.pone.0202758

**Published:** 2018-08-23

**Authors:** M. Pastorek, P. Muller, P. J. Coates, B. Vojtesek

**Affiliations:** Regional Centre for Applied Molecular Oncology, Masaryk Memorial Cancer Institute, Brno, Czech Republic; Université de Genève, SWITZERLAND

## Abstract

Response of tumours to Hsp90 inhibitors is highly variable and their clinical effects are unpredictable, emphasising the need for a predictive marker. We postulated that sensitivity to Hsp90 inhibitors is connected to basal proteotoxic stress that makes cells dependent on Hsp90. Therefore, we assessed HSF1 as a general sensor of proteotoxic stress and correlated its activity with sensitivity to three separate small molecule Hsp90 inhibitors in seven breast cancer cell lines representing each of the different cancer subtypes. Flow cytometry was used to analyse the viability of breast cancer cell lines after Hsp90 inhibition. HSF1 activity was characterised by Ser326 phosphorylation and the transactivation capacity of HSF1 was determined by qPCR analysis of the ratios of HSF1-dependent (HOP, Hsp70) and HSF1-independent (CHIP) chaperones and cochaperone mRNAs. We show that the sensitivity of breast cancer cell lines to Hsp90 inhibition is highly variable. The basal levels of phosphorylated HSF1 also vary between cell lines and the magnitude of change in HSF1 phosphorylation after Hsp90 inhibition showed a negative correlation with sensitivity to Hsp90 inhibitors. Similarly, the basal transactivation capacity of HSF1, determined by the ratio of Hsp70 or HOP mRNA to CHIP mRNA level, is directly proportional to sensitivity to Hsp90 inhibitors. Increasing basal HSF1 activity by prior heat shock sensitised cells to Hsp90 inhibition. These results demonstrate that endogenous HSF1 activity varies between individual cancer cell lines and inversely reflects their sensitivity to Hsp90 inhibitors, suggesting that basal proteotoxic stress is an important and generalised predictor of response. Mechanistically, the data indicate that high endogenous proteotoxic stress levels sensitise to Hsp90 inhibition due to the inability to respond adequately to further proteotoxic stress. HSF1 activity therefore represents a potential biomarker for therapy with Hsp90 inhibitors, which may be useful for the rational design of future clinical studies.

## Introduction

Hsp90 is a key component of the molecular chaperone system that cancer cells require to maintain activated oncoproteins including amplified/mutated membrane receptors, oncogenic kinases and transcription factors [1–3]. Hsp90 is highly active in cancer cells, which may be due to over-expression in some cancers [4–6] and/or its presence in a highly active multichaperone complex with increased ATPase activity [7, 8]. Our work also revealed that the assembly of Hsp90 is different in cancer cells due to phosphorylation that provides an enhanced pro-folding environment by modifying Hsp90’s interactions with its co-chaperones [9]. For these reasons, cancer cells show enhanced sensitivity to Hsp90 inhibitors compared to normal cells, allowing the ongoing development and clinical testing of Hsp90 inhibitors for cancer therapy [1–3]. On the other hand, patient response is highly variable and it has been suggested that sensitivity is associated with specific oncogenic or tumour suppressor proteins (e.g., HER2, ALK, EGFR, BRAF or p53) that are dependent on Hsp90 activity [3, 10, 11]. The presence or absence of these particular driver oncoproteins would therefore be predictive for patient response to Hsp90 inhibitor therapy. In addition, it has been noted that cancer cells suffer from proteotoxic stress due to their high levels of proteosynthesis and have to cope with metabolic stress, oxidative stress and hypoxia [12] and the enhanced antitumour effects of combining Hsp90 and proteasome inhibitors suggest that proteotoxic stress is a key determinant of Hsp90 inhibition success [13].

Proteotoxic stress leads to activation of the heat shock response that involves upregulation of chaperone expression and is always associated with enhanced activity of chaperones [14]. The heat shock response is itself regulated by the transcription factor HSF1, that binds to heat shock response elements (HREs) of genes that encode chaperones and co-chaperones, that in turn maintain protein folding activities. Therefore, we assessed the endogenous stress response of cancer cells by measuring HSF1 activity in correlation with sensitivity to Hsp90 inhibitors. Originally derived from natural products Geldanamycin and Radicol, current Hsp90 inhibitors are based mostly on purine scaffold or resorcyclic pyrazoles and bind to the ATP-binding pocket of Hsp90 [1–3]. We used three chemically distinct Hsp90 inhibitors to distinguish the principal mechanisms of sensitivity from pharmacokinetic effects.

## Materials and methods

### Cell cultures

The cell lines come from repositories of Masaryk Memorial Cancer Institute. The validity of cell lines was checked by sequencing, mycoplasma contamination was excluded by PCR test. All cell lines were obtained from American Type Culture Collection (ATCC, Manassas, VA). Human breast cancer cell lines BT-20 (ATCC® HTB-19™), BT-474 (ATCC® HTB-20™), BT-549 (ATCC® HTB-122™), MCF-7 (ATCC^®^ HTB-22^™^), MDA-MB-468 (ATCC® HTB-132™) and T-47D (ATCC® HTB-133™) were cultured in D-MEM, and SK-BR-3 (ATCC® HTB-30™) in McCoy’s medium, each supplemented with 10% fetal bovine serum and pyruvate [15–21]. Cells were grown to estimated 70% confluence before treatment.

### Viability assay

Cells were treated with Hsp90 inhibitors NVP-AUY922, BIIB021 and AT-13387 at final concentrations of 5, 10, 20, 50, 100, and 200 nM for 72 h. MCF-7 cells were additionally treated with 0.2, 0.5, 1, 2 and 5 μM NVP-AUY922 and BIIB021 for 72 h. To compare the sensitivity of cells cultivated in elevated temperature, SK-BR-3 and T-47D cells were cultured at 37 °C or at 39 ˚C in addition to NVP-AUY922 treatment for 72 h.

Cells were trypsinised and harvested in PBS and live cells were labelled with fluorescein diacetate (FDA) (Sigma, USA) for 25 min in the dark at room temperature and necrotic cells labelled with TO-PRO®-3 iodide (Thermo Fisher Scientific, USA). Stained cells were measured using a FACSVerse flow cytometer (BD Biosciences, USA) and data analysed by FCS Express version 4.0 (De Novo Software, USA). The effect of inhibition was calculated by subtracting the percentage of viable cells in treated samples from the percentage of viable cells in the corresponding control untreated sample.

### SDS-PAGE and immunoblotting

Total proteins (10 μg) were separated by SDS–polyacrylamide gel electrophoresis (SDS–PAGE) and transferred onto nitrocellulose membranes (Bio-Rad, USA). The blotted membranes were blocked in 5% milk and 0.1% Tween 20 in PBS for 1 h at room temperature and probed overnight with specific antibodies.

### Analysis of HSF1 phosphorylation

10^6^ cells were treated with 200 nM NVP-AUY922 for 4 h. Cells were washed twice in ice-cold PBS and lysed in buffer containing 0.5% CHAPS, 150 mM NaCl, 50 mM HEPES pH 7.5 and complete protease inhibitor and phosphate inhibitor cocktails (Sigma-Aldrich, USA). Samples were subjected to SDS-PAGE and immunoblotting, blots were incubated with anti-HSF1 mAb (SC-17756; Santa Cruz Biotechnology, USA), and anti-HSF1 (phospho S326) (ab76076; Abcam, USA). Signal from primary antibodies was detected using goat-anti rabbit IRDye® 800CW (92532211, LI-COR, USA) and Alexa Fluor® 647 labelled goat-anti mouse (Human adsorbed) (1010–31, Southern Biotech, USA) and analysed on a typhoon FLA 9500 laser scanner (GE Healthcare, USA) using ImageQuantTM software (GE Healthcare, USA).

### qRT-PCR

10^6^ cells were treated with 20 nM NVP-AUY922 for 24 h. Total RNAs were extracted using RNeasy Mini Kit (74106, Qiagen, USA) and 1 μg was reverse transcribed with random hexamer oligonucleotides using RevertAid First Strand cDNA Synthesis Kit (K1622, Thermo Fischer Scientific, USA). Triplicate samples were subjected to quantitative PCR analysis using SYBR Green Master (Roche, Switzerland) and 7900HT Fast Real-Time PCR system (Applied Biosystems, USA). Primer pairs used were Hsp70i_F 5'-gagtcctacgccttcaacat-3', Hsp70i_R 5'-gtgctcaaactcgtccttct-3', HOP_F 5´-ccccaggcactcagcgaacact-3´, HOP_R 5´-cagcggcggcacaaacagc-3´, CHIP_F 5´-gagaatggctgggtggaggactac-3´, CHIP_R 5´-ctcagcccagcccaccctcacct-3´, ActB_F 5´-gccgacaggatgcagaaggag-3´, ActB_R 5´-ctagaagcatttgcggtggac-3´. Absolute mRNA expression levels obtained using the standard curve method were normalised to the levels of β-actin. Note that the Hsp70 primers detect all major cytoplasmic Hsp70 isoforms [14].

## Results

### Breast cancer cell lines have variable response to Hsp90 inhibition

**Fig 1:**
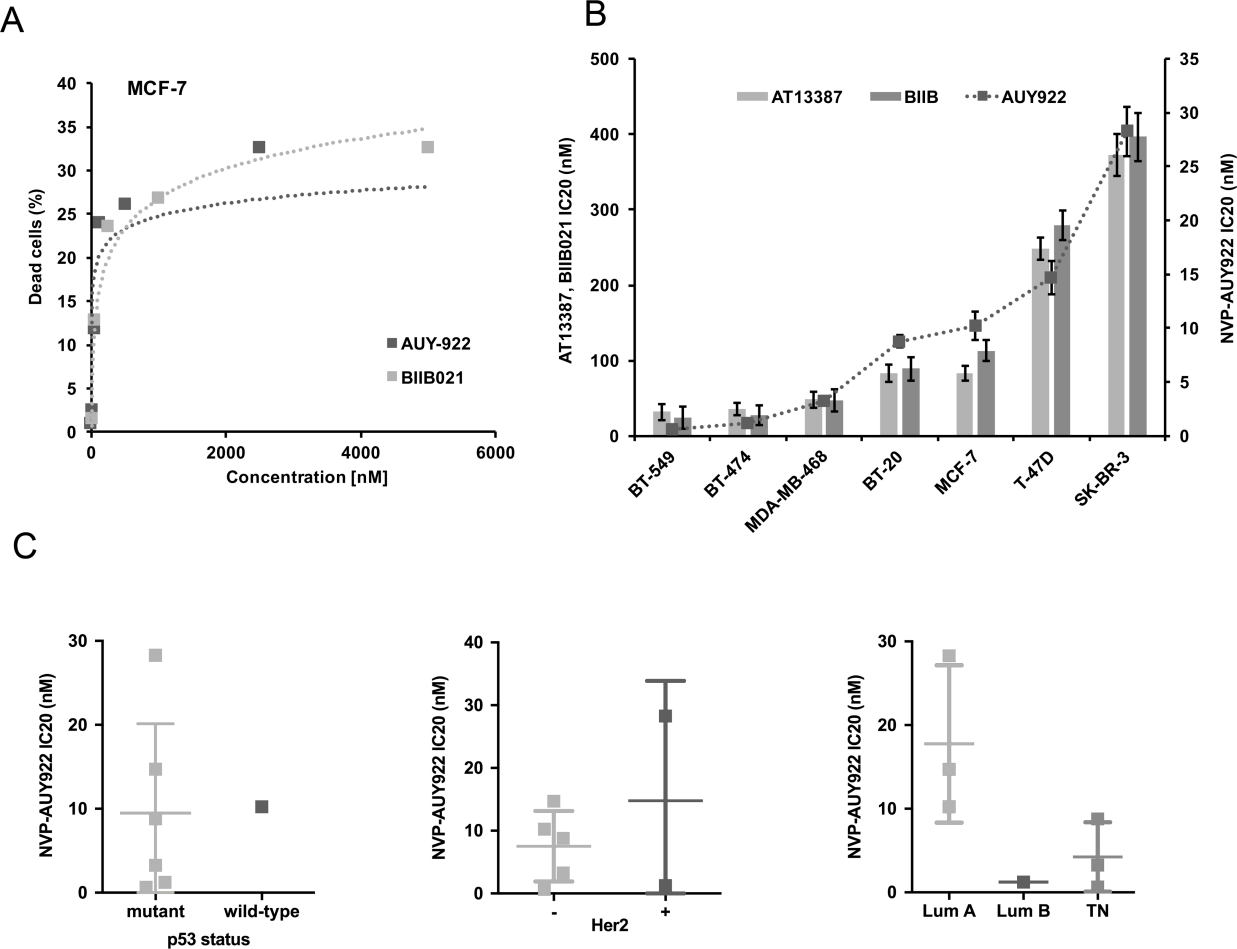
Analysis of cell viability after 72 h treatment with Hsp90 inhibitors NVP-AUY922, BIIB021 and AT-13387. (A) Toxicity of NVP-AUY922 and BIIB021 in MCF7 cells did not reach 50% in 72 h. All experiments were repeated 4 times. (B) Comparison of IC20 of Hsp90 inhibitors in breast cancer cell lines. (C) The comparison of sensitivity based on p53 status, Her2 (ErbB2) status, and subtype of breast cancer.

To investigate the potential of HSF1 as a predictive marker for Hsp90 inhibition, we selected seven breast cancer cell lines representing Luminal A (MCF-7, T-47D), Luminal B (BT-474), basal (BT-549, BT-20, MDA-MB-468) and HER2 positive (BT-474, SK-BR-3) subtypes. Sensitivity was measured using fluorescein diacetate (FDA) in combination with flow cytometry to identify live cells. Cells were treated for 72 h with N-terminal Hsp90 inhibitors that are based on either purine scaffold (BIIB021) or resorcylic pyrazoles (NVP-AUY922 and AT13387) with AT13387 currently undergoing clinical trials.All of these inhibitors mimic the structure that ATP adopts in the N-terminal nucleotide-binding pocket of Hsp90 [3]. We therefore expected that the effect of these inhibitors will not differ substantially in their mechanism of action, but may differ in the concentration needed to achieve the same level of inhibition. Our analysis revealed large differences in sensitivity of breast cancer cell lines to Hsp90 inhibition. The effect of Hsp90 inhibition on cell viability was not linearly dependent on concentration of the inhibitors and some cell lines reach a plateau prior to 50% reduction in cell viability (see Fig 1A for MCF-7 cells) (S1 Fig). Since further increases in inhibitor concentration did not result in higher toxicity over a 72 h period of exposure, we chose IC20 values to compare sensitivity of different cell lines. Our results show that the order of cell lines treated with NVP-AUY922 from most sensitive to most resistant was BT-549>BT-474>MDA-MB-468>BT-20>MCF-7>T-47D>SK-BR-3 (Fig 1B). Importantly, despite differences in absolute IC20 values, the relative sensitivity to all three inhibitors was broadly similar across the cell lines, suggesting that they are inherently different in their sensitivity to Hsp90 inhibition, with minimal effects due to pharmacogenetics of other off-target considerations (Fig 1B). Albeit our data are derived from a relatively small series of cell lines, IC20 values did not correlate with HER2, p53 wild type status, or luminal/triple negative status (Fig 1C).

### Characterisation of HSF1 activity

**Fig 2:**
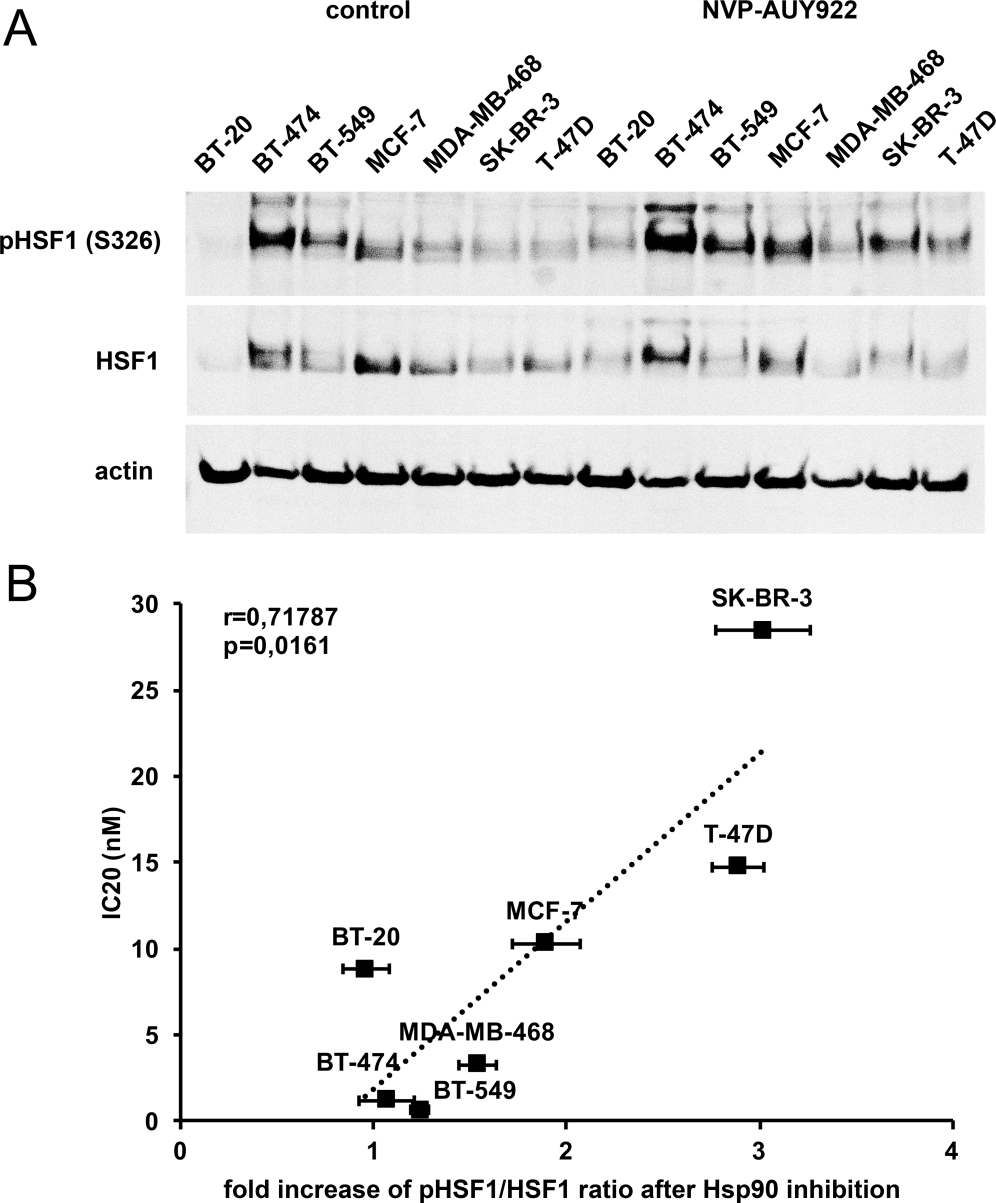
Analysis of HSF1 in response to NVP-AUY922. (A) Western blot analysis of phosphorylated HSF1 (Ser326) and total HSF1 in breast cancer cell lines. A representative blot is shown of 3 biological replicates. (B) Graph shows increase in ratio of pSer326HSF1 to total HSF1 after 4 h treatment with 200 nM NVP-AUY922 correlated to IC20 (nM) values of NVP-AUY922 treated breast cancer cell lines (r = 0.718; p = 0.0161).

**Fig 3:**
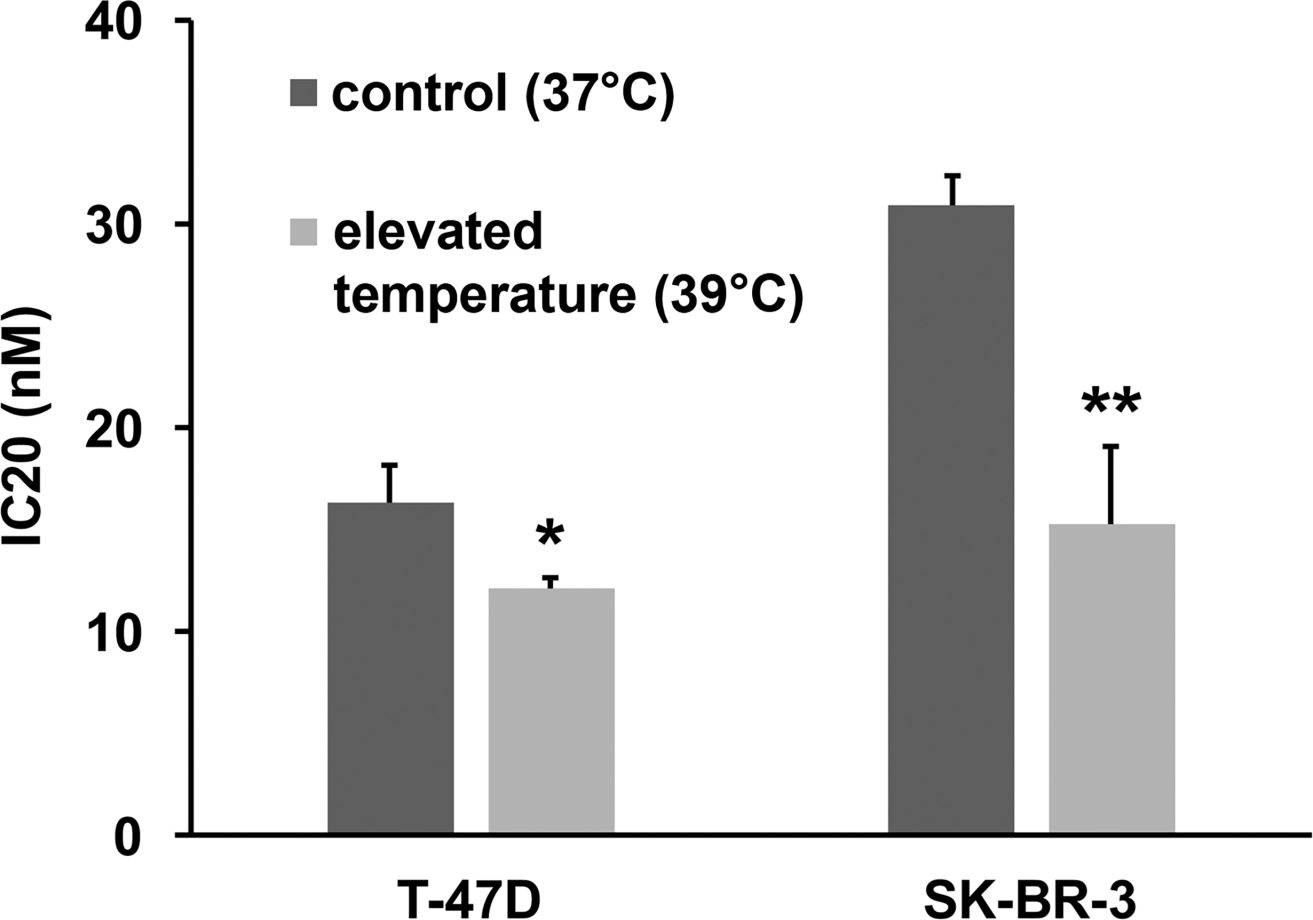
Viability analysis of SK-BR-3 and T-47D cells. SK-BR-3 and T-47D cells were cultivated in physiological conditions (37 °C) and elevated temperature (39 °C) after 72 h NVP-AUY922 treatment. Viability analysis was performed in 3 biological replicates. (* p = 0,0377; **p = 0,0023).

Since over- or under-expression of Hsp90 is seen in a variety of tumour types, sometimes in association with gene amplification or loss, and has prognostic impact [4–6, 22, 23], Hsp90 levels could themselves be of predictive value for Hsp90 inhibitor therapy. However, recent data from phase I/II trials of various solid tumour types found that Hsp90 levels are not a predictive biomarker for Hsp90 inhibitor response [24]. In this respect, the activity of Hsp90 in cancer cells is highly dependent on other chaperones and co-chaperones like AHA1, p23, HOP (also known as STIP1), CHIP (STUB1), Cdc37, Hsp40 and Hsp70 [25, 26]. Like Hsp90 itself, many of the pro-folding co-chaperones are transcriptionally regulated by HSF1, a major response factor to proteotoxic stress [27], leading us to hypothesise that HSF1 activity, as a marker of proteotoxic stress, may act as a predictive factor for Hsp90 inhibition. HSF1 is present in an inactive state in the cytoplasm in unstressed cells and translocates to the nucleus when activated by stress, where it acts as a DNA-bound transcription factor [27, 28]. To characterise HSF1 activity we analysed HSF1 phosphorylation on serine 326, a post-translational modification that is not necessarily required for HSF1 activation following stress, but appears to be a good marker of activation [27, 28] (Fig 2A) (S1 Image). Quantitative analysis showed that the increase in ratio of phosphorylated HSF1 to total HSF1 after NVP-AUY922 treatment correlates with the resistance of breast cancer cell lines to Hsp90 inhibition (r = 0.718; p = 0.0161) (Fig 2B) (S2 Fig). We also observed that cells most resistant to Hsp90 inhibition do not contain activated, phosphorylated HSF1 under normal growth conditions, as opposed to the most sensitive cell lines. We therefore selected the most resistant breast cancer cell lines (SK-BR-3, T-47D), that also show the highest increase in Ser326 phosphorylation of HSF1 upon Hsp90 inhibition, and cultivated them in elevated temperature (39 °C) to increase the demands on chaperone system. Analysis of viability after 72 h showed that cells cultivated at 39 °C were significantly more sensitive to Hsp90 inhibition compared to cells cultured at 37 °C (Fig 3) (S3 Fig). Since subjecting the cells to cultivation in conditions with increased temperature had no effect on their viability, we infer that their decreased survival upon Hsp90 inhibition is connected to pre-activation of HSF1 by increased temperature, leading to an inability of cells to further increase HSF1 activity after Hsp90 inhibitor treatment.

### HSF1 activity defined by HOP/CHIP ratio correlates with sensitivity to Hsp90 inhibition

**Fig 4:**
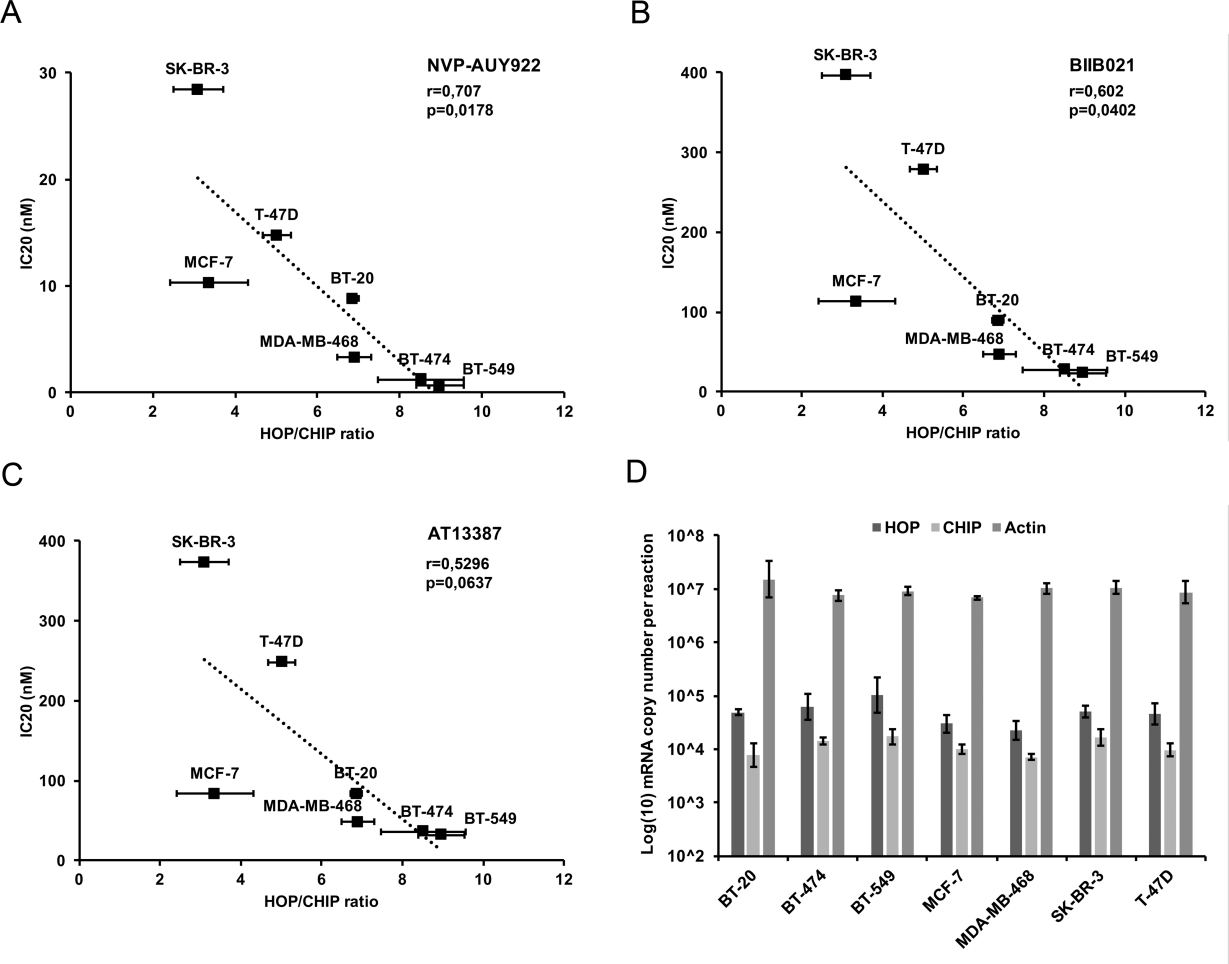
Correlation of HSF1 basal activity characterised by HOP/CHIP ratio with sensitivity to Hsp90 inhibitors. (A-C) HSF1 activity expressed as the ratio between the HSF1 regulated co-chaperone HOP and the HSF1 independent co-chaperone CHIP correlated with IC20 (nM) of the indicated Hsp90 inhibitors. (A) NVP-AUY922 (r = -0.707; p = 0.0178), (B) BIIB021 (r = -0.602; p = 0.0402) and (C) AT13387 (r = -0.530; p = 0.0637). (D) mRNA copy number of analysed genes per reaction. qPCR measurements were performed from 3 biological replicates.

As another approach to measuring HSF1 transcriptional activity, we also performed qPCR to analyse mRNA levels of the co-chaperones HOP (encoded by *STIP1*) and CHIP (encoded by *STUB1*) in stressed and unstressed conditions (Fig 4) (S4 Fig). HOP (Hsp Organizing Protein) is a co-chaperone assisting in forming a complex between Hsp70 and Hsp90 [29, 30] whereas CHIP (Carboxyl-terminus of Hsp70-Interacting Protein) is an E3 ubiquitin ligase that regulates ubiquitination and subsequent protein degradation of chaperone clients and tumour-related proteins. Although both of these proteins are part of a chaperone complex with Hsp70 and Hsp90, the gene encoding HOP contains a consensus Heat Shock Element (HSE) and is tightly regulated by HSF1, whereas the CHIP encoding gene does not contain an HSE element and is independent of HSF1 [29, 31]. Thus, the ratio of mRNA levels coding HOP and CHIP should reflect HSF1 transcriptional activity. Additionally, as opposed to other genes coding for the major chaperones regulated by HSF1 (Hsp70, Hsp90, Hsp40 etc.), HOP is present as a single copy gene and can therefore serve as a more precise marker of HSF1 transcriptional activity. Our results show that basal HSF1 activity defined by HOP/CHIP ratio in unstressed cells negatively correlates with IC20 values of cell lines to Hsp90 inhibitors after 72 h treatment, with correlation coefficients of -0.707 for NVP-AUY922 (p = 0.0178), -0.602 for BIIB021 (p = 0.0402) and -0.5296 for AT13387 (p = 0.0637).

**Fig 5:**
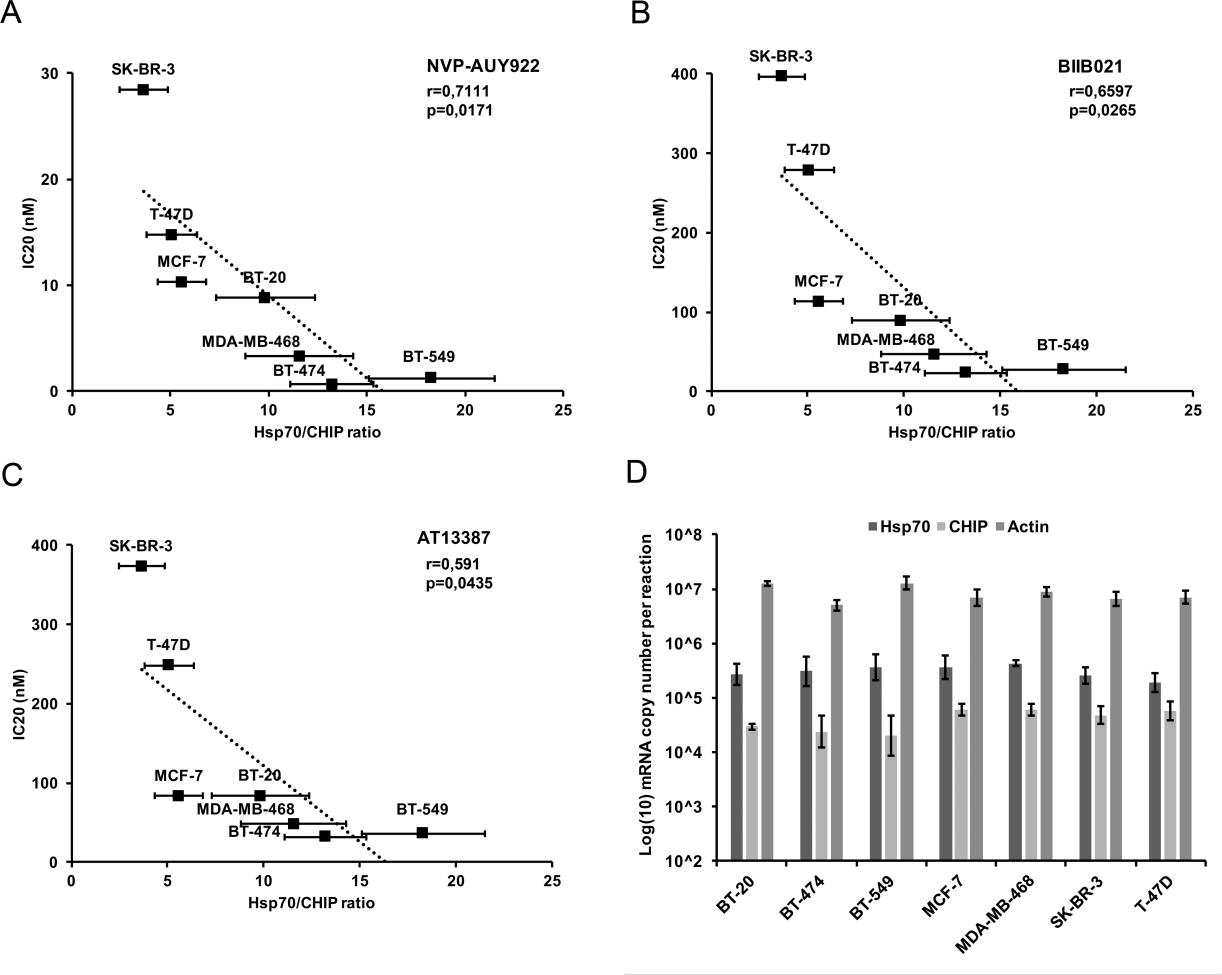
Correlation of HSF1 basal activity with sensitivity characterised by Hsp70/CHIP ratio to Hsp90 inhibitors. (A-C) HSF1 activity expressed as the ratio between the HSF1 regulated co-chaperone Hsp70 and the HSF1 independent co-chaperone CHIP correlated with IC20 (nM) of the indicated Hsp90 inhibitors. (A) NVP-AUY922 (r = -0.711; p = 0.0171), (B) BIIB021 (r = -0.660; p = 0.0265) and (C) AT13387 (r = -0.591; p = 0.0435). (D) mRNA copy number of analysed genes per reaction. qPCR measurements were performed from 3 biological replicates.

To confirm these results, we also performed qPCR analysis of HSF1 regulated isoforms of Hsp70 and characterised basal HSF1 activity in unstressed cells by the Hsp70/CHIP mRNA ratio (Fig 5) (S5 Fig). In concordance with the previous results, basal activities of HSF1 defined by Hsp70/CHIP ratio also negatively correlate with the IC20 values of breast cancer cell lines to Hsp90 inhibitors, with correlation coefficients of -0.7111 for NVP-AUY922 (p = 0.0171), -0.6597 for BIIB021 (p = 0.0265) and -0.591 for AT13387 (p = 0.0435). As an independent verification, we retrieved RNA-Seq data from 675 cancer cell lines [32]. We retrieved levels of HSF1-regulated chaperones for the studied breast cancer lines and compared with relative sensitivity. These data showed a similar pattern as our RT-PCR data, with a high ratio of inducible Hsp70 (*HSPA1A*)/CHIP mRNA showing an inverse correlation (-0.9429; p = 0.00480) with cell survival. Similarly, Grp78 (*HSPA5*), Hsp60 (*HSPD1*) and Hsp110 (*HSPH1*)/CHIP ratios showed correlations of –0.8286, p = 0.0416; -0.7714, p = 0.0724; and -0.8286, p = 0.0416, respectively. In contrast, the constitutive Hsp70 isofom Hsc70 (HSPA8)/CHIP ratio showed no correlation (-0.3143, p = 0.5441) (S1 Dataset).

## Discussion

Hsp90 inhibitors have shown promise for cancer treatment and their efficacy is the subject of ongoing clinical trials. Hsp90 inhibition shows high selectivity for cancer cells, with minimal side-effects on normal tissues [1–3]. To date, it is clear that some patients benefit, whilst others do not, a problem exemplified by our demonstration of plateau effects in some cell lines–patients with these types of tumours are unlikely to show any benefit. Thus, there is a need for predictive biomarkers, but the only strong indicator of response in a series of Phase I/II trials appears to be ErbB2 [24], whereas other markers including Hsp90, Hsp70, EGFR and steroid receptors were not predictive. This contrasts with the observations that triple-negative breast cancers (that lack ErbB2) may show a good response to Hsp90 inhibitors [33]. In our data, albeit derived from a limited number of cell lines, ErbB2 amplification also showed no association with sensitivity to three different Hsp90 inhibitors. Thus, whilst ErbB2 may be a target for Hsp90 inhibition, other important factors exist that influence sensitivity.

Hsp90 inhibition leads to HSF1 activation and in turn to enhanced expression of HSF1-inducible chaperones and co-chaperones, which compensate for the effect of Hsp90 inhibitors [34]. Recent data demonstrated that cancers can be divided into two groups depending on their “epichaperome”, with a more complex chaperome indicating sensitivity to Hsp90 inhibition [35]. Those data were interpreted as indicating that the complexity of the chaperome is an indicator of the reliance of tumour cells on Hsp90. Turning this notion on its head, we therefore hypothesised that the complexity of the epichaperome will itself be determined by the major regulator of the chaperome, HSF1. Indeed, our findings indicate that cells with high basal transcriptional activity of HSF1 are more sensitive to Hsp90 inhibitors. This activity was measured by both a known activating post-translational modification (Ser326 phosphorylation) and by transcriptional activation of two downstream chaperone/co-chaperone target genes compared to a non-HSF1 target co-chaperone gene.

Our data therefore indicate that the relative disturbance in endogenous proteotoxic stress differs in different cancers and is reflected in the basal activity of HSF1, a main regulator of chaperone expression. Introduction of additional external proteotoxic stress by Hsp90 inhibition then leads to further demands on chaperone activities that need to be met by increased transcriptional activity of HSF1. As opposed to cells with low basal HSF1 activity, cells that already possess high levels of active HSF1 have a more limited capacity to further activate HSF1 and are unable to proportionally respond. This hypothesis is supported by the finding that the most resistant cell lines (with low endogenous HSF1 activity and therefore higher capacity to respond to Hsp90 inhibition) become more sensitive to inhibition of Hsp90 by prior exposure to increased temperature. It is also possible that differential sensitivity to Hsp90 inhibition reflects the amount of inhibitor that accumulates within the different cell lines. However, in turn, the affinity of Hsp90 to its inhibitors and their accumulation within cells is dependent on Hsp90 activity [8], which is itself regulated by proteotoxic stress. Thus, variations in intracellular Hsp90 inhibitor levels and HSF1 are likely to correlate with each other.

In conclusion, our data show that assessment of endogenous proteotoxic stress, measured by endogenous HSF1 activity serves as a marker of cancer cell sensitivity to Hsp90 inhibition. The data are similar to other recent data that analysed chaperome complexity as a predictive biomarker [35] and suggest that HSF1 may provide a more generalised marker than the analysis of individual specific driver oncogenic events (such as ErB2, p53, BRAF, EGFR, ALK etc [3, 10, 11] in patient tumours, where other factors are likely to provide confounding effects. In particular, this approach may provide a relatively simple, rapid and cost-effective predictive biomarker for cancer patients that will benefit most from Hsp90 inhibitor therapy.

## Supporting information

S1 Dataset Supplementary dataset for correlation of cell viability with RNA-Seq data. (XLSX)

S1 Fig Analysis of cell viability after 72 h treatment with Hsp90 inhibitors NVP-AUY922, BIIB021 and AT-13387. (XLSX)

S2 Fig Analysis of HSF1 in response to NVP-AUY922. (XLSX)

S3 Fig Viability analysis of SK-BR-3 and T-47D cells. (XLSX)

S4 Fig Correlation of HSF1 basal activity characterised by HOP/CHIP ratio with sensitivity to Hsp90 inhibitors. (XLSX)

S5 Fig Correlation of HSF1 basal activity with sensitivity characterised by Hsp70/CHIP ratio to Hsp90 inhibitors. (XLSX)

S1 Image Sample of original WB image Alexa 647 and IRD 800 dye. (ZIP)
