# Supplementary figures and images for: Intrinsic proteotoxic stress levels vary and act as a predictive marker for sensitivity of cancer cells to Hsp90 inhibition
Source: PLoS One. 2018 Aug 23;13(8):e0202758. doi: 10.1371/journal.pone.0202758 (PMC6107219; doi:10.1371/journal.pone.0202758)

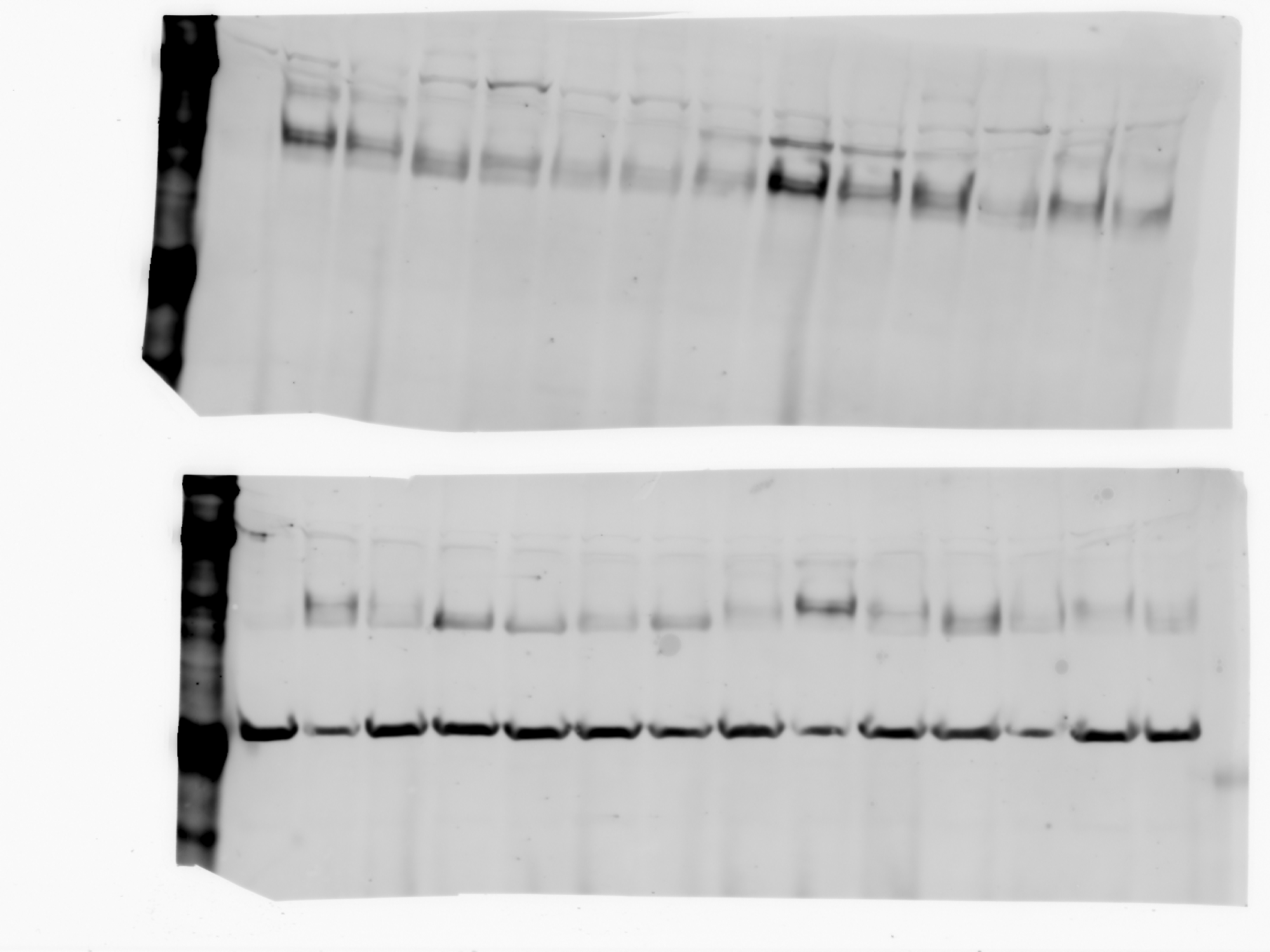

Supplement: S1 Image — (ZIP) [file pone.0202758.s007.zip › S1_Image/HSF1(2nd gel), pHSF1(1st gel,1st row), act(2nd gel,2nd row).tiff]

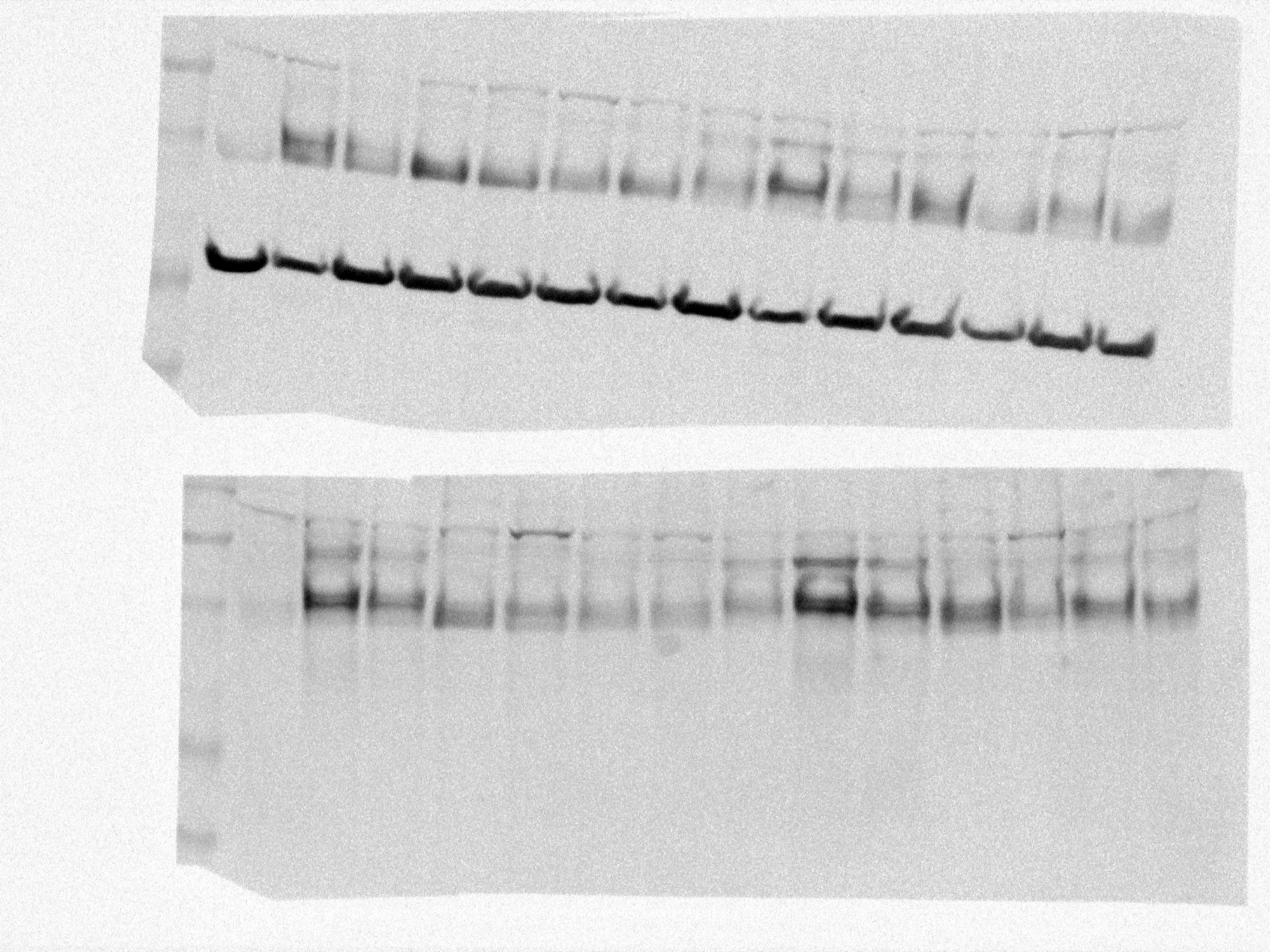

Supplement: S1 Image — (ZIP) [file pone.0202758.s007.zip › S1_Image/HSF1(1st gel,1st row), pHSF1(2nd gel), act(1st gel,2nd row).tiff]
